# Mathematical Modeling of the Lethal Synergism of Coinfecting Pathogens in Respiratory Viral Infections: A Review

**DOI:** 10.3390/microorganisms11122974

**Published:** 2023-12-13

**Authors:** Ericka Mochan, T. J. Sego

**Affiliations:** 1 Department of Computational and Chemical Sciences, Carlow University, Pittsburgh, PA 15213, USA; 2 Department of Medicine, University of Florida, Gainesville, FL 32611, USA; timothy.sego@medicine.ufl.edu

**Keywords:** influenza A virus, coinfection, immune response, mathematical modeling

## Abstract

Influenza A virus (IAV) infections represent a substantial global health challenge and are often accompanied by coinfections involving secondary viruses or bacteria, resulting in increased morbidity and mortality. The clinical impact of coinfections remains poorly understood, with conflicting findings regarding fatality. Isolating the impact of each pathogen and mechanisms of pathogen synergy during coinfections is challenging and further complicated by host and pathogen variability and experimental conditions. Factors such as cytokine dysregulation, immune cell function alterations, mucociliary dysfunction, and changes to the respiratory tract epithelium have been identified as contributors to increased lethality. The relative significance of these factors depends on variables such as pathogen types, infection timing, sequence, and inoculum size. Mathematical biological modeling can play a pivotal role in shedding light on the mechanisms of coinfections. Mathematical modeling enables the quantification of aspects of the intra-host immune response that are difficult to assess experimentally. In this narrative review, we highlight important mechanisms of IAV coinfection with bacterial and viral pathogens and survey mathematical models of coinfection and the insights gained from them. We discuss current challenges and limitations facing coinfection modeling, as well as current trends and future directions toward a complete understanding of coinfection using mathematical modeling and computer simulation.

## 1. Introduction

Influenza A virus (IAV) infection is a major yearly global health burden. A typical seasonal influenza infection is a mild, survivable viral infection. However, IAV infection is regularly complicated by coinfection with either a second virus or with a bacterium, leading to increased morbidity and mortality [1]. The likelihood of these coinfections increases in influenza pandemics, further exacerbating the morbidity and associated costs of the epidemic [2,3,4].

IAV–bacteria coinfection is most often associated with *Streptococcus pneumoniae* (pneumococcus) and *Staphylococcus aureus,* though coinfections with other bacteria strains such as *Haemophilus influenzae* and *Streptococcus pyogenes* are also regularly diagnosed [2,5,6,7]. By some estimates, around 75% of hosts infected with influenza that eventually develop pneumonia have a confirmed bacterial coinfection [1]. The devastating severity of the 1918 influenza pandemic is thought to be largely attributed to widespread secondary bacterial infections, particularly with pneumococcus [2,8,9]. About 90% of samples tested from fatal IAV infections were positive for at least one bacterial coinfection [2]. Analyses of samples from patients fatally infected with the 2009 H1N1 pandemic also indicated a prevalence of secondary bacterial infections, particularly from pneumococcus or methicillin-resistant *S. aureus* (MRSA) [4]. Influenza is thought to change the lung epithelium in a way that creates an environment suitable for pneumococcal pneumonia, which may turn a typical, seasonal influenza infection into a severe or even fatal coinfection [10,11,12,13].

Coinfection with a second respiratory virus has also been identified as a major contributor to morbidity and mortality in both influenza and coronavirus pandemic years [14,15]. With the advent of more sophisticated testing techniques, patients tested for respiratory viruses for diagnoses have been increasingly shown to exhibit more than one virus in the lungs at the same time. By some estimates, 10% to 55% of respiratory viral infections involve more than one virus [16,17,18,19,20], though some reports estimate this value to be as high as 70% [21]. Viruses such as IAV, coronavirus, rhinovirus, and respiratory syncytial virus (RSV) tend to be co-circulating in the population and may readily infect the same vulnerable respiratory tract. Each of these viruses may also be paired with a variety of bacterial strains, resulting in a high number of possible combinations of virus–bacteria coinfection, each with its own dynamics and symptoms [22].

Despite its prevalence, much remains unknown about the clinical impact of coinfection. In some studies, coinfections have been shown to be substantially more fatal than single infections [23,24,25,26], while in other studies, coinfection did not play a major role in determining the fatality of the infection [27,28]. The complicated, interconnected dynamics of these coinfections make it difficult to experimentally determine the impact of each individual pathogen on the coinfected host. Variability in host species, pathogen species, and experimental conditions such as inoculum or timing further complicate the studies of coinfection, making it challenging to generalize IAV coinfection.

Modeling of biological phenomena is an important and growing field in mathematics. Models allow us to identify and analyze key mechanisms in the immune response to infection. Models can be used to quantify elements of the intra-host immune response that are difficult to obtain and measure experimentally. In silico experimentation is vital to expanding our knowledge of the immune response and the mechanisms by which pathogens may evade it. Improving and increasing the library of available models for superinfection is a crucial next step in mathematical biology.

Currently, there are many published models of IAV single infection or bacterial single infection, but not nearly as many models of coinfection dynamics. In this review, we describe the current state of the literature in modeling the immune response to viral and bacterial respiratory infections, and the gaps in knowledge that modeling may endeavor to fill.

## 2. Virus–Bacteria Coinfections: Immune Dysregulation and Mathematical Models

### 2.1. Changes and Dysregulation of the Immune Response after Virus–Bacteria Coinfection

Virus–bacteria coinfections generally cause increased morbidity and mortality for the host, regardless of the two pathogens causing the infection. Multiple theories exist as to which mechanisms are primarily responsible for this increased lethality [29,30,31,32,33,34,35,36,37,38], but no single theory adequately explains the multiple interconnected changes that occur to the immune system during coinfection. Cytokine dysregulation, changes to immune cell activation and function, mucociliary dysfunction, and alterations to the respiratory tract epithelium have all been identified as causes for increased lethality after coinfection. The relative importance of each of these mechanisms varies with the type of pathogens, timing and sequence of infections, inoculum size, and other experimental conditions.

#### 2.1.1. Dysregulation of Cytokine Responses

The pro-inflammatory cytokine response is dysregulated by viral infections and is believed to contribute to the increased severity of secondary bacterial coinfection [39]. Pro-inflammatory cytokines are released soon after the viral infection begins in the lung. These pro-inflammatory cytokines promote the upregulation of platelet-activating factor receptor (PAFR), which pneumococcus can use to invade the epithelium and instigate secondary bacterial infection [32].

Many studies report significantly increased pro-inflammatory cytokine and chemokine concentrations after secondary bacterial infection [38,40,41,42]. However, several studies have also shown substantial decreases in certain cytokines and chemokines related to effector cell function. For example, the production of neutrophil chemoattractants such as KC (CXCL1) and MIP2 (CXCL2) is diminished after secondary bacterial infection [43]. This lack of chemoattractants can exacerbate the infection by limiting the innate immune response to both pathogens. However, in some cases, minimizing inflammatory responses is actually advantageous to the host. For example, decreased levels of IL-6 have been shown to increase survival from infection [44]. These complex dynamics and lack of generalization about the immune response further complicate our understanding of the cytokine dynamics in virus–bacteria infection.

Interferons (IFNs) are critical to the antiviral immune response, as they inhibit viral replication and activate adaptive immune responses. A strong and prompt IFN response is crucial to controlling a viral infection. However, there is evidence that interferon also reduces antibacterial host responses in secondary bacterial infections. Type I IFN (IFN-α/β) has been shown in multiple experiments to decrease antibacterial responses within the lung in response to both *S. aureus* and pneumococcus [43,45,46,47].

Type II IFN (IFN-γ) also plays a complex role in the antiviral and antibacterial defenses in the host respiratory tract. IAV infection in IFN-γ-deficient mice produced less inflammation and disease severity [48]. In mice, IAV decreased immunity to pneumococcus by reducing IFN-γ-dependent proliferation of memory T-helper cells and migration of CD4+ T cells [49]. Decreased IFN-γ levels have been shown to be advantageous for host survival [44,50].

To date, not many studies have focused on the impact of type III interferon (IFN-λ) on morbidity and mortality in superinfection. IFN-λ weakens bacterial clearance in IAV–MRSA and IAV–pneumococcus superinfection [51]. In mice, IFN-λ produced by dendritic cells as an antiviral measure leads to epithelial damage and increased likelihood of secondary bacterial infection [52].

Anti-inflammatory cytokines also exhibit interesting and complex behaviors in the wake of secondary bacterial infection. IL-10 has been identified as a potential key player in the dysregulation of cytokine activity in coinfection, especially when there is a time delay between the initiation of the two pathogens. IL-10 production is stimulated after an infection to control the inflammatory response, but if a secondary infection begins after inflammatory responses have been halted, that secondary infection can become deadly. IL-10 inhibits antibacterial defenses, and treatment of IAV-infected mice with anti-IL-10 antibodies can improve survival rates from secondary bacterial infection [38]. Transforming growth factor (TGF)-β is another anti-inflammatory cytokine released after the onset of viral infection. The influenza neuraminidase activates latent TGF-β, which can diminish inflammatory responses for the subsequent bacterial infection [53].

#### 2.1.2. Changes to Effector Cells’ Activation and Function

Closely tied to the changes to cytokine expression are the activation, recruitment, and function of immune effector cells. Alveolar macrophages (AMs) are the first line of phagocytic defense against lung pathogens. A robust macrophage response to infection is key to controlling pathogen replication. AM responses are dysregulated in the presence of influenza [54] and their clearance of bacterial pathogens is inhibited by IAV through poorly understood mechanisms [55]. IAV has also been shown to inhibit the recruitment of macrophages in mice, resulting in both enhanced pneumococcus colonization and susceptibility to pneumonia caused by *S. aureus* [11]. In the first few days after virus infection, AMs exhibit strong phagocytic responses, but later experience weakened phagocytic ability [30]. Sun and Metzer found that IFN-γ suppresses AM activity post-influenza infection, again leading to a weakened resistance to pneumococcal infection [30]. Neutralizing the IFN-γ response can reestablish the bacterial clearance rates.

Neutrophil responses are also altered after coinfection. Neutrophils are a major component of innate immune responses and are particularly important for early clearance of bacterial infection. Multiple studies have shown that the initial recruitment of neutrophils in response to secondary bacterial infection is decreased [38,43,56,57], though some studies show this recruitment returns to normal levels a few days post-bacterial infection. In addition to diminished early recruitment, neutrophils also often become less effective in the wake of secondary infection [40,56,58]. IAV infection has been shown to decrease neutrophil phagocytosis and the generation of reactive oxygen species, leading to greater susceptibility to pneumococcus [59]. These weakened responses can thus make it easier for bacteria to replicate and cause a severe infection.

#### 2.1.3. Effect of Damage to the Epithelium

Viral infections have been shown to alter the epithelium such that epithelial cells become more permissive to the adherence of bacteria [10,32]. Epithelial cell death by IAV infection promotes bacterial invasion and colonization by upregulating bacterial adhesion [60]. Pneumococcus pathogenesis is enhanced by epithelial damage from IAV infection [61], which exposes cryptic binding sites [62] and the basement membrane [63] or disrupts barrier function potentially leading to pneumococcus outgrowth, dissemination, and invasion into the bloodstream [64]. Alveolar barrier disruption during coinfection has been primarily attributed to the dysregulation of the host immune response [12].

Lethal synergism between IAV and pneumococcus leads to decreased rates of repair of epithelial damage, further enhancing morbidity and mortality due to coinfection [65]. Exposure of undifferentiated airway epithelial cells during wound healing provides adhesion sites for *S. aureus* [66]. Damaged epithelium may also disrupt mucociliary clearance, allowing the bacteria to replicate more quickly within the lung tissue. In an in vivo study of murine tracheal tissue, the influenza virus did not increase the adherence of bacteria to the epithelium, but rather decreased the velocity of mucociliary action and thus the clearance rate of bacteria [67]. Other respiratory viruses, such as rhinovirus and RSV, in addition to bacterial and fungal pathogens, have all been shown to reduce mucociliary clearance in lung tissue [68]. This sustained disruption of nonspecific clearance mechanisms further enhances the likelihood of subsequent respiratory infections.

#### 2.1.4. Effect of Timing and Sequence of Inocula

Timing between infections and types of infections has been shown to be a major determinant of the severity of infection as well. For example, McCullers et al. [32] showed that infection with influenza A virus 7 days before pneumococcus infection was lethal in 100% of mice tested, whereas infection with pneumococcus bacteria 7 days before influenza A infection was 100% survivable. Mice singly infected with influenza only or bacteria only showed some lethality, indicating the host defenses were somehow enhanced when bacteria were administered first. The same has been shown in mice for coinfection with IAV and MRSA, where the infection was most pathological and lethal when MRSA was administered 0–3 days after IAV [25].

When bacteria are the first pathogen to invade the respiratory tract, there may be different effects on the immune response than when the virus is first. For example, pneumococcal nasopharyngeal colonization in mice resulted in impaired IFN-α production and higher viral load during IAV infection [69].

### 2.2. Mathematical Modeling of Dynamics of Respiratory Virus–Bacteria Coinfection

#### 2.2.1. Within-Host Ordinary Differential Equation (ODE) Models of Coinfection

Despite many models published on respiratory virus-only [70,71,72,73,74,75,76,77,78] and bacteria-only [79,80,81,82,83] infections, few ODE models currently exist that can replicate influenza–bacteria coinfection dynamics [42,44,84,85,86,87,88,89]. The majority of ODE modeling work in coinfection has been done in IAV and pneumococcus coinfection, one of the most common coinfections diagnosed in a typical influenza season [14].

There are some commonalities between these various ODE models, primarily in the structure of terms controlling the innate immune response to infections. Existing models are largely target cell-limited models, which model the target epithelial cells as a limited resource within the lung environment that cannot be replenished. Most of the models also include terms for alveolar macrophages, specifically emphasizing the importance of a robust and functioning macrophage population to limit the likelihood of a lethal secondary bacterial infection. A few models include terms for cytokines, such as interferon or TNF-α, or other immune cells, such as neutrophils or B cells, but most models are comparatively small and concise. These models provide a strong foundation upon which more complex models may be built, to further our understanding of the dynamics of the immune response to coinfection. Table 1 summarizes the ODE models of virus–bacteria coinfection included in this review.

In one of the first published models of influenza–pneumonia coinfection, Smith et al. designed a small, target cell-limited model to explore the mechanisms involved in the replication and survival of each pathogen in the presence of the other [84]. Their work explores the hypothesis that macrophage function may be impaired following influenza infection, leading to decreased bacterial clearance and thus increased lethality of influenza–pneumonia coinfection. They also hypothesize that the presence of bacteria may lead to an increased rate of viral release from the infected epithelial cells. These hypotheses cannot be confirmed via mathematical modeling, though the authors do suggest possible experimental techniques to test the theories. Their model also includes terms representing mechanisms for increased epithelial cell death due to pneumococci as well as increased bacterial adherence and carrying capacity in the presence of a virus, but these terms are less influential in the overall behavior of the model.

Smith and Smith also expanded upon this work by exploring the effect of the bacterial inoculum on the likelihood of instigating a secondary bacterial infection [85]. As before, the model emphasizes that alveolar macrophages are critical to controlling the onset of bacterial infection, and depleting these macrophages decreases the inoculum of pneumococci required to cause the secondary infection. The extent and severity of macrophage depletion varies throughout the course of a typical influenza infection, and thus this dependence on the macrophage population may explain why the likelihood of a severe secondary bacterial infection also varies with time post-viral infection.

Building upon the Smith model, Duvigneau et al. explored a variety of mechanisms by which bacterial clearance rates may be impaired after influenza infection [42]. By calibrating their model to each of several candidate cytokines, the authors tested hypotheses on which cytokines are most influential to the diminished bacterial clearance rates often seen in the wake of influenza infection. Type II interferon (IFN-γ) had the greatest effect on the bacterial clearance rates, but TNF-α and IL-6 on their own did not exhibit much negative effect on the clearance rates. These authors later built upon their work to show, through data-driven experimentation, that neutralizing IFN-γ improves bacterial clearance in the lungs [44]. Schmit et al. have also shown experimentally that IFN-γ is a major contributor to the lung damage experienced during influenza infection, and mice without IFN-γ demonstrate increased resistance to secondary bacterial infection [48]. Sun and Metzger also verified that IFN-γ impedes macrophage function, which can make a host more susceptible to a secondary bacterial infection [30].

Cheng et al. worked with the Smith model to study the dynamics of coinfection, specifically within the inflammatory response [86]. Their investigations showed the most severe inflammation would occur with bacteria administered seven days after influenza infection. These results align with those found experimentally by McCullers et al. [32]. Boianelli et al. also expanded upon the Smith model to test antiviral treatment regimens and their effect on coinfection dynamics [89].

In contrast to the previous models, Shrestha et al. built an ODE model of influenza–pneumonia coinfection [88] based on the within-host model of influenza infection dynamics by Handel et al. [90]. The authors explored the effect of timing and inoculum on the likelihood of survival of coinfection. This model demonstrates that bacteria administered 4–6 days post-influenza infection can initiate a serious secondary infection with a much lower inoculum than in hosts without a pre-existing viral infection. The model also shows the importance of providing any antiviral treatment within 4 days post-infection to prevent the host’s defenses from weakening enough to allow the secondary bacterial infection to take hold. As in the previous models, macrophages are the primary phagocytic cells in the model used to control the infections.

**Figure 1:**
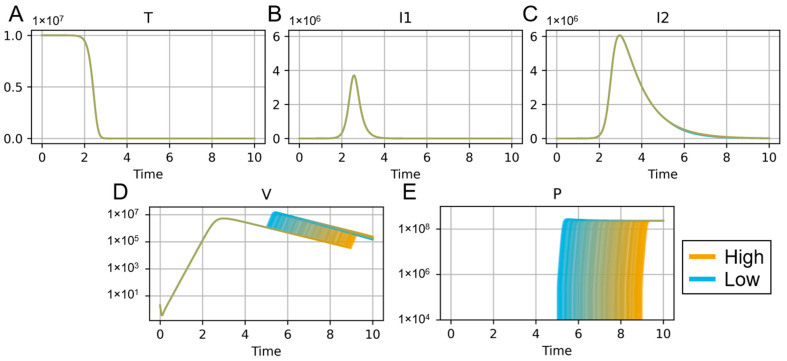
Simulation of IAV–streptococcus coinfection according to the model in Smith and Smith [85] varying time of secondary infection. Target cells (“T”, panel (**A**)) that become infected enter an eclipse phase (“I1”, panel (**B**)), transition to producing IAV (“I2”, panel (**C**)), and are eventually cleared. IAV (“V”, panel (**D**)) is administered at time 0. Streptococcus (“P”, panel (**E**)) is administered at varying times of secondary infection. Time is shown in units of days. The time of secondary infection was uniformly sampled between times 5 and 9 for 1000 replicates. The inset shows colors for the time of secondary infection, from time 5 (“Low”) to time 9 (“High”).

We demonstrate the versatility of these in-host mathematical models in Figure 1, which simulates results from the Smith and Smith model [85]. In this simulation, we vary the time at which the bacterial infection is initiated, relative to the initial viral infection. This model always leads to total depletion of the target epithelial cells (T) and infected cells (I1 and I2). The virus (V) reaches its peak about 3 days post-infection and then begins to decline as the target cell population is eradicated. The virus experiences a rebound after the secondary bacterial infection is initiated (P). We simulated 1000 iterations of this model, uniformly sampling a distribution of the time of administration of bacteria, between 5 and 9 days post-infection. While the time at which the virus rebounds is dependent upon the onset of the secondary pathogen, the magnitude and duration of the rebound are consistent regardless of the time the bacteria enter the host.

#### 2.2.2. Population-Level Dynamics of Virus–Bacteria Coinfection

Virus–bacteria coinfections have also been modeled with population-level models. Shrestha et al. used longitudinal data from influenza seasons to quantify the likelihood of bacterial pneumonia after influenza infection and found that influenza infection increases the risk of pneumonia by about 100-fold [91]. Other models have been proposed to identify mitigation strategies for post-influenza pneumonia. Some studies indicate that, due to the high likelihood that influenza strains will develop a resistance to antiviral measures as an epidemic progresses, antibacterial interventions may be important to reduce morbidity and mortality in a pandemic [92]. Widespread implementation of mitigation strategies such as social distancing or vaccination may be effective in reducing the spread of multiple circulating pathogens [93]. The effectiveness of antiviral or antibacterial measures in a population is also likely to be highly strain-dependent [94].

## 3. Virus–Virus Coinfection: Viral Competition and Mathematical Models

Viral coinfections commonly present in both children [17,95,96,97,98,99] and adults [17,100,101] with acute respiratory infection. Millions of children are afflicted with lethal respiratory infections each year, particularly in Africa and Asia [99]. RSV and rhinovirus are commonly found in young children; RSV infects about 90% of children within their first two years of life [102], and rhinovirus is commonly detected in even asymptomatic children [96,103]. While these two viruses are not often found simultaneously in a single host, their coinfection does cause prolonged respiratory symptoms [96]. As with virus–bacteria coinfections, the strain of virus, timing between infection, and in-host immune response to the two infecting viruses will impact the dynamics and symptoms of the viral coinfection in the host.

### 3.1. Changes and Dysregulation of the Immune Response after Virus–Virus Coinfection

#### 3.1.1. Interferon Stimulation and Antiviral Immunity

Much of the work done studying immune dysregulation in virus–virus coinfection has been in interferon stimulation and avoidance by viruses. When the first virus invades the host, it stimulates an interferon response by the host to slow viral replication and release. This interferon response often limits the ability of the second virus to replicate and cause infection as well [104,105,106,107]. In turn, viruses have evolved to develop evasion techniques to avoid the host’s antiviral defenses and allow the viral coinfection to persist.

As in virus–bacteria coinfections, the timing between the two viral inocula will greatly impact the likelihood of a successful defense against infection. If the host mounts a strong enough defense against the first virus, the immune system may be primed to defend against the second. However, the second virus may also cause an overstimulation of inflammatory responses, leading to cytokine dysregulation, excess symptoms, and damage to the host. The synergistic and antagonistic mechanisms by which two viruses interact are highly dependent on the individual species; even different strains of the same virus may react differently in coinfection [108,109].

#### 3.1.2. Resource Limitation and Competition

When two viruses simultaneously infect the same respiratory tract, there is a natural competition between pathogens for the limited resources within the tract. Some virus–virus interactions can result in more severe pathology for the host, while other interactions may prevent the second virus from productively infecting the host. These agonistic and antagonistic interactions of competing viruses impact the seasonality and circulating patterns of common viruses, such as influenza, rhinovirus, RSV, and coronavirus [108,110,111,112,113,114]. For example, a prominent circulation of rhinovirus likely delayed the onset of the 2009 influenza pandemic in many European countries [111,115]. The onset of rhinovirus was also shown to protect against SARS-CoV-2 infection [106].

The viruses are also likely competing for a limited number of cell surface receptors in order to infect epithelial cells lining the respiratory tract [116,117,118]. However, some viral pathogens have been shown to enhance the availability of target receptors of other viruses, such as increased expression of ACE2, the target receptor of SARS-CoV-2, through stimulation of IFNr [119] by influenza independently of IFN [120].

### 3.2. Mathematical Modeling of Dynamics of Virus–Virus Coinfection

#### 3.2.1. Within-Host ODE Models of Virus–Virus Coinfection

A few ODE models of virus–virus coinfections have also been published in recent years, as experimental evidence has shown the widespread prevalence of dual virus infections in patients admitted with influenza-like infections. Table 2 summarizes the models of virus–virus coinfection discussed in this section.

Despite the prevalence of multi-virus infections, a limited number of papers have modeled dual virus coinfection in the respiratory tract. Pinky and Dobrovolny have studied the dynamics of multiple viruses replicating and competing for resources within a single respiratory tract in several papers [121,122,123,124]. Competition for the limited resources within the epithelium, specifically the availability of epithelial cells to infect, is a major contributor to the dynamics of the two coinfecting viruses [121]. Through experimental and simulated data of influenza A, RSV, rhinovirus, parainfluenza, and hMPV, the authors determined swift and sustained viral replication is paramount for one virus to survive in the presence of another; one virus may drive out the other by being first to infect the host’s target epithelial cells. Simulations indicated the virus with the fastest replication rate (rhinovirus) was able to weaken the replication of other viruses in its vicinity, whereas the slowest replicating virus (parainfluenza) was largely overwhelmed by the other, quicker viruses [121]. Viruses that infect first tend to get a head start on the trailing virus, and they have a greater likelihood of successfully competing for the limited resources available in the host’s respiratory tract. In several follow-up studies, these authors further explored the dynamics of cell regeneration [122,124], stochasticity [123], and dosage levels [126] on the replication and viability of two competing viruses.

**Figure 2:**
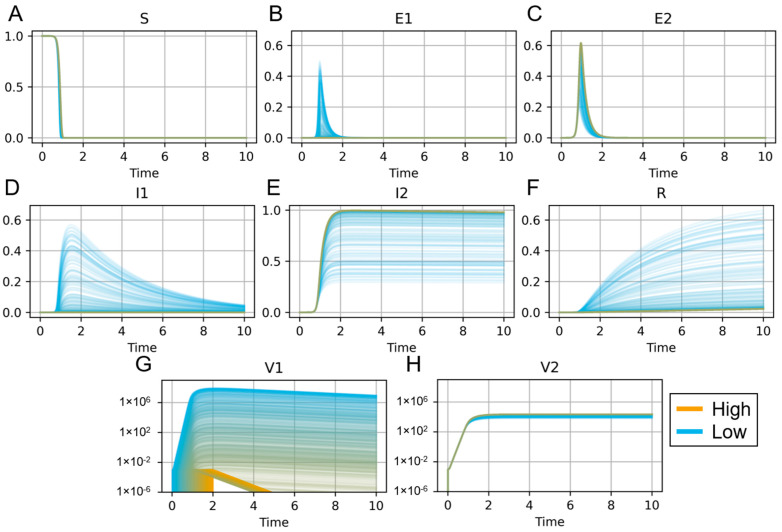
Simulation of IAV–RSV coinfection according to the model in [121] varying the time of secondary IAV infection. Susceptible uninfected cells (“S”, panel (**A**)) are infected by either IAV (“V1”, panel (**G**)) or RSV (“V2”, panel (**H**)), enter an eclipse phase (“E1”, panel (**B**), for IAV, “E2”, panel (**C**), for RSV), become productively infectious (“I1”, panel (**D**), for IAV, “I2”, panel (**E**), for RSV), and then die (“R”, panel (**F**)). RSV is administered at time 0. IAV is administered at varying times of secondary infection. Time is shown in units of days. The time of secondary infection was uniformly sampled between times 0 and 2 for 1000 replicates. The inset shows colors for the time of secondary infection, from time 0 (“Low”) to time 2 (“High”).

In Figure 2, we demonstrate the dynamics of a virus–virus coinfection model [121] when varying the time at which the secondary infection is initiated. In these simulations, the initial RSV infection (V2) is administered at day 0, and the onset of the secondary influenza infection (V1) is taken from a uniform distribution between 0 and 2 days. We again performed 1000 replicates of these simulations to indicate the sensitivity of the model variables to the timing between infections. When the influenza is administered within a day of the initial RSV infection (blue lines), the secondary infection is detrimental to the host. The influenza titers (V1) remain elevated long after the initial infection, and many more cells die (R). When there is more time between infections (yellow lines), the influenza titers remain low and clear quickly, indicating the secondary infection will not adversely affect the host, and the RSV would have won the competition for limited resources within the host’s lung environment.

As with virus–bacteria coinfections, the timing between subsequent viral infections is highly influential to the dynamics of the infections [125]. Cao et al. explored this timing in a series of mathematical models calibrated to data from coinfected ferrets [127]. Like the previous models, these experiments showed that the first virus to infect and replicate in the epithelium can delay and/or block the second virus from infecting the host. As different virus strains instigate the innate immune response, specifically the interferon response, at different rates, the order and timing between the subsequent virus infections has a major impact on the overall likelihood of survival of the host.

#### 3.2.2. Population-Level Models of Virus–Virus Coinfection Dynamics

While within-host models give a picture of the immune response of one individual against one or more infections, population-level models can yield further insights into the transmissibility, infectivity, and severity of epidemics within a population of susceptible individuals. ODE modeling of epidemics has been studied for about a century, starting with the most basic Susceptible-Infected-Recovered (SIR) model. Only recently have these SIR models expanded to include multiple pathogens within one epidemic. Most of these models also test the effects of immunity within some members of the population as well, either through a treatment regimen or through existing natural immunity to the pathogen.

Recently, a number of studies have endeavored to model transmissibility, virulence, and time-dependent dynamics in a population-level model of coinfection [110,128,129]. Merler et al. created a modified SIR model that features SIR dynamics for a pandemic strain of influenza and SIS dynamics for a co-circulating secondary respiratory virus, such as rhinovirus [128]. These multidimensional dynamics indicate that circulating rhinovirus can increase the transmissibility of influenza, leading to potential multiple waves in the influenza season dynamics. Chen et al. also modeled multiple circulating viruses in a population following SIS dynamics, in which infected individuals may become susceptible again [129]. Nickbakhsh et al. further studied co-circulating viruses in a population to explain temporal dynamics in a typical rhinovirus and IAV season [110].

## 4. Current Limitations in Coinfection Modeling

Mathematical modeling of coinfection currently contends with several limitations that inhibit its utility in understanding the complex interactions between a host and multiple pathogens. Generally, there is a significant lack of longitudinal data with sufficient time resolution to confidently perform model development and selection and produce a well-calibrated model. This lack of data, which is perpetuated by the expense and difficulty of collecting the experimental data, also manifests in limited measures of host health and immune response, which inhibits establishing relationships between datasets from different experiments and thus constructing a model that integrates the collective body of published experiments. As such, most modeling applications in coinfection, to date, have been limited to describing the dynamics of marginal pathological and immunological processes compared to the immense complexity of the total interactions of a host and multiple pathogens. Integrative models that describe the complexity of the overall immune response (e.g., by incorporating multiple datasets) can further the development of a comprehensive quantitative description such as those demonstrated by projects such as FIRM [130], GranSim [131], and ImmSim [132,133].

Spatial models such as those implemented in projects such as GranSim have been developed at a wide variety of model scales and targeted a diverse set of applications, such as the spatial distribution and spread of virions [134], local recruitment and effects of the immune response [135], and contagion cooperation in host populations [136]. To our knowledge, no in-host model exists that describes influenza–bacteria coinfection dynamics with any significant emphasis on spatial dynamics and pathogen specificity. However, several recently developed spatial and multiscale models could lay the groundwork for explaining spatially resolved observations of influenza–bacteria coinfection at one or multiple scales. Of interest are multicellular spatial agent-based models (ABMs), which consider the interactions of individual cells and/or pathogens (i.e., the agents) while modeling cell shape and motility, spatial distributions, and transport of biomolecular species, and other multicellular-level objects and processes. Spatial ABMs are especially well suited for modeling tissue complexity and heterogeneity because they provide a natural framework for describing the properties and processes of pathogens and cells on the basis of their state and local environment. Spatial ABMs have described the cellular and spatial aspects of viral infection comparably to population models [137], and recently in simulations of complex, heterogeneous tissues on the order of millions of cells [138].

**Figure 3:**
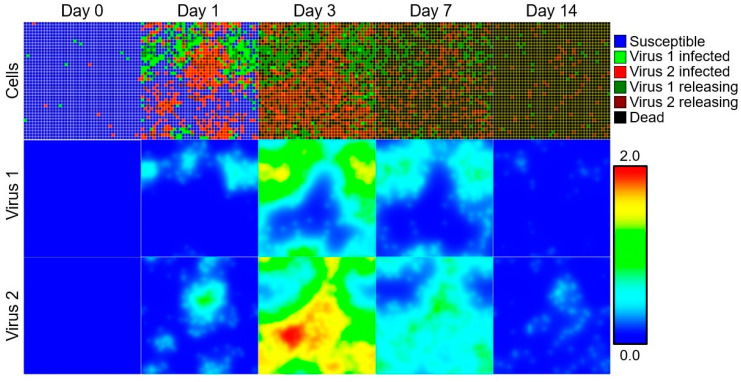
Two-dimensional, spatial simulation of viral–viral coinfection according to the infection model in Figure 2 [121]. The agent-based multicellular spatial model and model parameters were taken from [139]. The simulation consists of a field of individual cells (“Cells”, **top**) and two viruses represented as diffusive fields (“Virus 1”, **middle**, and “Virus 2”, **bottom**). Susceptible cells are infected by either Virus 1 or Virus 2, enter an eclipse phase (“Virus 1 infected” for Virus 1, “Virus 2 infected” for Virus 2), become productively infectious (“Virus 1 releasing” for Virus 1, “Virus 2 releasing” for Virus 2), and then die (“Dead”). Results are shown for days 0 (**left**), 1 (**left-center**), 3 (**center**), 7 (**right-center**), and 14 (**right**). Cell types are shown in the top-right legend. Virus concentrations are shown according to the bottom-right color bar. The simulation was implemented in CompuCell3D [140].

We demonstrate the potential utility of these spatial models with the simulations in Figure 3. We simulated the virus–virus coinfection model presented in Figure 2, but now allowing spatial effects to impact the dynamics of the coinfection in the host. Using parameters from previous work [139], we demonstrate the time- and space-dependent dynamics of the competing viruses in the host. At day 0, the majority of cells are considered susceptible (blue) and both viruses exist at very low levels. As the infection progresses through days 1 and 3 post-infection, the virus populations grow, and the number of infected cells increases. We see distinct patterns of virus growth in the tissue: cells affected by a given virus (green or red cells) tend to be in close proximity to one another. One to two weeks post-infection, the epithelium is comprised largely of dead or productively infected cells, and the virus levels begin to diminish as they run out of new cells to infect. Accounting for the spatial heterogeneity in coinfections will be key to future mathematical modeling of these infections.

Recent ABMs of viral infection have described host–pathogen interactions with increasingly greater simulation detail and model complexity by coupling multicellular ABMs with descriptions of subcellular state, viral kinetics, and organismal-level immune response. One recent ABM demonstrated the importance of timing in antiviral therapies by considering a local distribution of susceptible tissue, where each susceptible cell was modeled with an embedded ODE model of viral replication that was calibrated to SARS-CoV-2 data, and each embedded ODE was coupled with local diffusive transport of extracellular virus [141]. Several methodologically similar ABMs have since described other viral pathogens, contexts, and applications. One ABM described the spatial dynamics of influenza plaque growth by embedding an ODE model of the JAK/STAT pathway and interferon signaling in susceptible cells [142]. Another ABM showed the role of cellular drug metabolic variability in promoting the spread of SARS-CoV-2 by coupling subcellular and multicellular models with PK/PD modeling of drug dosing [143]. An investigation of the basic biological and cellular aspects of immune response to influenza infection generated another ABM from a calibrated ODE model of influenza infection and innate and adaptive immune responses [144].

While recent work has made progress toward quantitatively describing the cellular- and tissue-specific aspects of infection and host immune response, a quantitative model of coinfection with local specificity, especially virus–bacteria coinfection, remains out of reach. Some cell-based spatial models of local infection have delivered promising frameworks for describing the interplay between subcellular, tissue, and host dynamics. However, most efforts have focused on viral infection and there is a general lack of quantitative models to describe most bacterial pathogens such as *S. aureus* and pneumococcus with sufficient detail to develop a comprehensive model of a particular microenvironment during viral–bacterial coinfection and immune response. Such models could generate and/or test hypotheses related to the interplay between pathogens or enhanced pathogenicity through manipulation of the local microenvironment and immune response. While ODE modeling is an important first step to understanding these complicated dynamics, ODE models cannot account for the spatial detail that is likely critical to understanding how those dynamics produce observed viral plaques and bacterial colonies in the respiratory tract. Some ODE models attempt to account for spatial and resource limitations by creating target cell-limited models that limit the pathogen through exhaustion of resources, but these models inaccurately describe the reality of target organs and tissues such as the airway epithelium and overall health of the host. Detailed models of heterogeneous tissues and specific microenvironments can consider the complex dynamics of target tissues such as those of the epithelium to identify the mechanisms and conditions of critically important events such as the spread of infection, onset of pneumonia, and organ failure.

## 5. Conclusions

In this review, we explored within-host and population-level mathematical models of respiratory virus–bacteria and virus–virus coinfection. We have emphasized some opportunities for further exploration and research, including more detailed and dynamic ODE models, as well as the incorporation of spatial information in the models. Coinfections present complicated, time-dependent dynamics, and these may best be investigated through mathematical modeling and in silico experiments to develop more effective interventions against coinfection.

## Figures and Tables

**Table 1:** Summary of models of virus–pneumonia coinfection.

Reference	Notable Variables and Parameters	Primary Results and Conclusions
Duvigneau et al. [42]	Interferon-γ	IFN-γ weakens bacterial clearance, allowing for increased post-influenza bacterial replication.
Sharma-Chawla et al. [44]	Interferon-γ IL-6	Neutralizing IFN-γ improves bacterial clearance. Neutralizing both IFN-γ and IL-6 further improves bacterial clearance after influenza infection.
Smith et al. [84]	φ (decreased rate of macrophage phagocytosis) ψ (increased bacterial carrying capacity) μ (increased bacterial adherence to epithelial cells)	Viral titers increased in the presence of bacteria, and post-influenza macrophage impairment allows bacteria to grow at a faster rate.
Smith and Smith [85]	Φ (percent of alveolar macrophage depletion)	Macrophage depletion, bacterial growth rates, and bacterial inoculum are interconnected, and balancing them is key to survival of the coinfection.
Cheng et al. [86]	TNF-α	TNF-α levels can reflect the overall level of inflammatory response, providing an early warning against possible cytokine storm.
Shrestha et al. [88]	Time between influenza and bacterial inoculation Bacterial inoculum size	Bacteria administered 4–6 days post-influenza produce the most severe infections and require a lower inoculum size than coinfections started outside of this window.

**Table 2:** Summary of models of virus–virus coinfection.

Reference	Notable Variables and Parameters	Primary Results and Conclusions
Pinky and Dobrovolny [121]	Size and timing of secondary viral inoculum	Primary viruses can block secondary viruses by infecting host cells without viral interference.
Pinky and Dobrovolny [122]	Cell regeneration rate	Chronic coinfection was not possible for the considered coinfection models with cellular regeneration. Only a single-virus infection could produce chronic infection.
Pinky et al. [123]	Relative viral production rate	Stochasticity allows a slower-growing virus to outcompete a faster-growing virus.
Pinky et al. [124]	Infection rate of superinfected cells Cell regeneration rate	Chronic viral coinfection required both cell superinfection and regeneration.
Cao et al. [125]	Rate of IFN-induced conversion from target cells to virus-resistant cells Viral production rate sensitivity to IFN Killing rate of infected cells by IFN-activated NK cells	Viral hierarchy could be reproduced with IFN inhibition of viral production and IFN-mediated killing of infected cells by NK cells. Viral hierarchy and interactions between competing viruses are highly dependent on the timing of secondary infection.
